# Supplementary material for: Glimepiride-Loaded Nanoemulgel; Development, In Vitro Characterization, Ex Vivo Permeation and In Vivo Antidiabetic Evaluation
Source: Cells. 2021 Sep 13;10(9):2404. doi: 10.3390/cells10092404 (PMC8467883; doi:10.3390/cells10092404)
Supplement: Supplementary file 1 [file cells-10-02404-s001.zip › cells-1266350-supplementary.pdf]

Supplementary materials:

Table S1. Evaluation of hypoglycemic effect of GMP and GMP/βCD/GEL-44/16 loaded nanoemulgels and oral glimepiride in streptozocin induced diabetes model. (Mean ± SD, *n* = 5).

Groups	0 h	2 h	4 h	6 h	8 h	10 h	12 h	24 h
1(Normal control)	100± 15.79	100.4± 13.61 ^{ns}	103.4± 13.84 ^{ns}	100.8± 11.51 ^{ns}	103.4± 14.29 ^{ns}	100.8± 10.37 ^{ns}	104.2± 15.51 ^{ns}	90.2± 12.23 ^{ns}
2(Diabetic control)	277.2± 13.95	274.6± 12.93^{ns}	275.8± 18.37^{ns}	267.8± 10.94^{ns}	271.2± 10.23^{ns}	269.6± 10.96^{ns}	261.8± 10.80^{ns}	265.4± 13.75^{ns}
3(Positive control)	260.4± 10.16	168.2± 12.31***	176.4± 14.38***	195± 15.41***	223.6± 13.34***	234.4± 12.70 ^{ns}	248.6± 14.25^{ns}	254.4± 12.13^{ns}
4(Blank gel formulation)	264.2± 11.73	260.2± 10.73^{ns}	242.6± 8.08***	223.6± 15.97***	201.8± 11.58***	207.3± 15.41***	219.8± 14.78***	248.4± 11.32^{ns}
5(GMP loaded nanoemulgel)	265.8± 11.60	248.2± 12.91^{ns}	232± 13.07**	208.2± 10.42***	165.4± 11.58***	170.6± 9.39***	186.4± 10.28***	201.8± 10.89***
6(GMP/βCD/GEL-44/16 loaded nanoemulgel)	266.8± 10.28	252.2± 10.49^{ns}	229.2± 14.44***	196.4± 11.80***	150.2± 15.61***	156.4± 10.33***	171.4± 17.34***	183.2± 8.61***

Values presented are Mean ± SD of five individual subjects (n = 5) where ns = p > 0.05, * = p < 0.05, ** = p < 0.01 and *** = p < 0.001. Black color shows the multiple comparison of blood glucose value at each time interval of group 1 verses its 0 h, blue color shows the multiple comparison between group 2, green color shows the multiple comparison between group 3, Purple color shows the multiple comparison between group 4, Yellow color shows the multiple comparison between group 5 and red color represents the comparison between group 6 correspondingly.

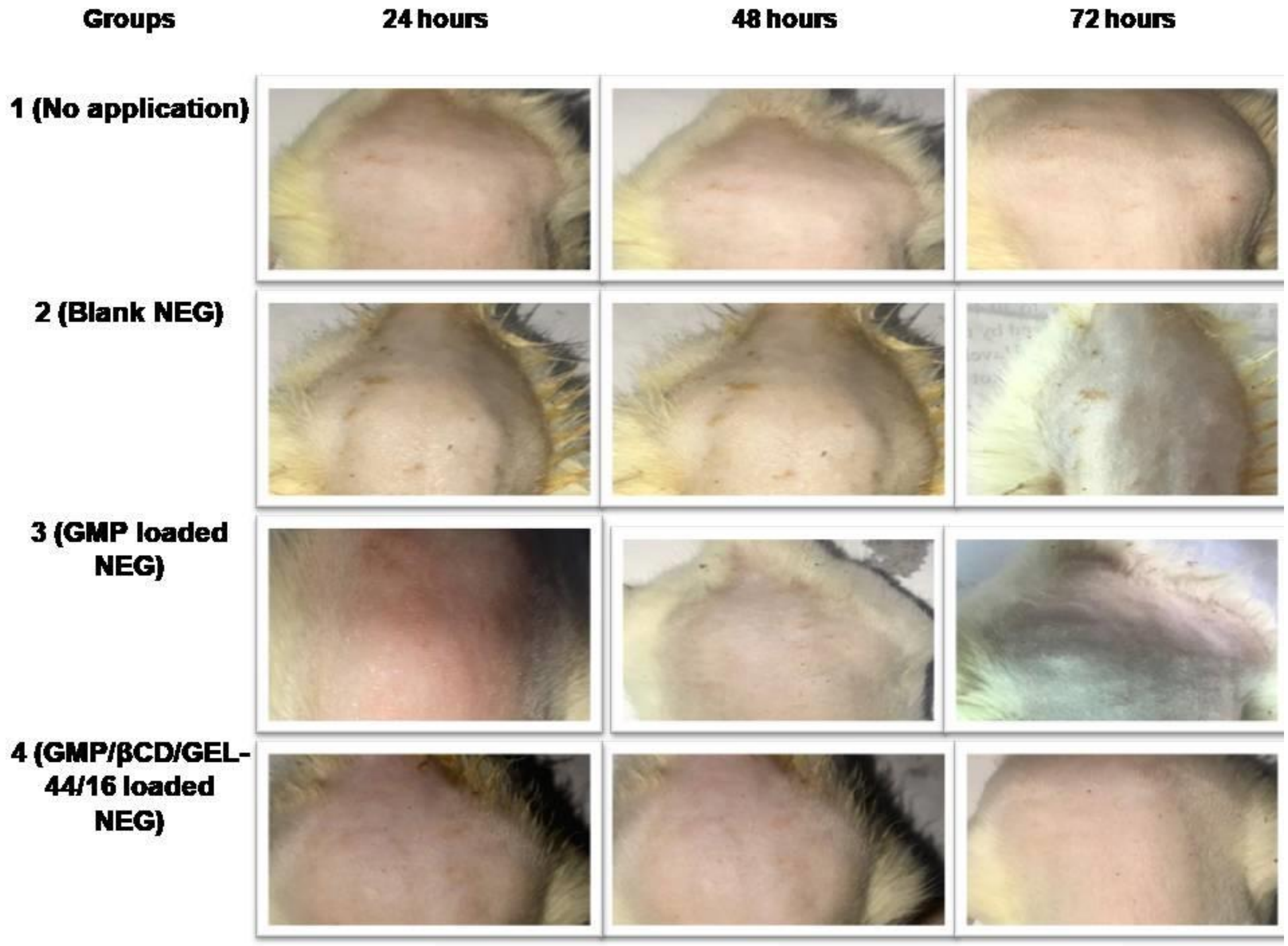

Figure S1. Shaved skin images of rats at different time of exposure.
